# Diagnostic Value of the Combination of Golgi Protein 73 and Alpha-Fetoprotein in Hepatocellular Carcinoma: A Meta-Analysis

**DOI:** 10.1371/journal.pone.0140067

**Published:** 2015-10-06

**Authors:** Meiyu Dai, Xiaoli Chen, Xuexiang Liu, Zheng Peng, Jie Meng, Shengming Dai

**Affiliations:** Department of Clinical Laboratory, The Fourth Hospital Affiliated to Guangxi Medical University, Liuzhou City, Guangxi Province, China; Yong Loo Lin School of Medicine, National University of Singapore, SINGAPORE

## Abstract

Conflicting results have been widely reported on the use of Golgi protein 73 (GP73) as a serum biomarker for diagnosing hepatocellular carcinoma (HCC). This study evaluated the accuracy of GP73, alpha-fetoprotein (AFP), and GP73 + AFP for diagnosing HCC. The meta-analysis was performed on 11 studies that were selected by means of a comprehensive systematic literature review. Summary diagnostic accuracy, meta-regression analysis for heterogeneity and publication bias, and other statistical analyses were performed using Meta-Disc (version 1.4) and Stata (version 12.0). Pooled sensitivity, specificity, and diagnostic odds ratio were 0.77 (95% CI: 0.75–0.79), 0.91 (95% CI: 0.90–0.92), and 12.49 (95% CI: 4.91–31.79) for GP73; 0.62 (95% CI: 0.60–0.64), 0.84 (95% CI: 0.83–0.85), and 11.61 (95% CI: 8.02–16.81) for AFP; and 0.87 (95% CI: 0.85–0.89), 0.85 (95% CI: 0.84–0.86), and 30.63 (95% CI: 18.10–51.84) for GP73 + AFP. The area under the curve values were 0.86, 0.84, and 0.91 for GP73, AFP, and GP73 + AFP, respectively. These results indicate that for HCC diagnosis, the accuracy of GP73 was higher than that of AFP, and that GP73 + AFP exhibited significantly higher diagnostic accuracy than did GP73 or AFP alone.

## Introduction

Hepatocellular carcinoma (HCC) is one of the most common malignant cancers and the third leading cause of cancer-related deaths worldwide among men aged between 40 and 59 years [1]. HCC prevalence is high in Asia and in western and central Africa [2]. In USA, the incidence of HCC increased during 1973–2011 on a year-on-year basis [3]. The 5-year recurrence rate of HCC is 48.8%, and the mean survival time is between 54.4 and 70.0 months [4]. A 10-year survey conducted in China indicated that the social cost and burden of HCC was the highest among chronic diseases listed by the WHO [5]. Therefore, early detection and effective treatment are crucial for improving the survival and quality of life of patients with HCC.

Since the 1970s, alpha-fetoprotein (AFP) has been used as a primary diagnostic serum biomarker of HCC. However, serum AFP is not an accurate biomarker of HCC because of its low sensitivity and specificity [6–9]. Therefore, a novel serum biomarker that exhibits superior diagnostic accuracy is required for diagnosing HCC. Recent studies have identified various new tumor biomarkers such as Golgi protein 73 (GP73, also known as GOLPH2), interleukin–6, and squamous cell carcinoma antigen. GP73, a Golgi type II transmembrane protein of unknown function, is expressed at low levels in biliary epithelial cells in healthy livers and is detected in human serum [10,11], and the expression of GP73 is upregulated in the hepatocytes of patients with viral and non-viral liver diseases [12]. Serum GP73 and AFP have been used as biomarkers of HCC in several studies, but the results of these studies are heterogeneous and conflicting [13–16]. The present study performed a systematic literature review and meta-analysis to evaluate the accuracy of serum GP73 + AFP for diagnosing HCC.

## Material and Methods

### Identification of studies

We comprehensively and systematically searched the literature for peer-reviewed English-language studies published in PubMed, Embase, or Web of Science before May 1, 2015. The keywords for the search included (1) GP73: GP73, Golgi protein 73, Golgi phosphoprotein 2, Golgi membrane protein 1; and (2) HCC: HCC, hepatocellular carcinoma, liver cancer, liver cell carcinoma, hepatic cell carcinoma. Furthermore, references of selected studies and other relevant published reports were manually searched. If more than one study was based on the same research topic or contained the same data, only the highest-quality study was selected. Conference abstracts and letters to the editor were excluded because these provided limited information.

### Selection criteria

The titles and abstracts of selected studies were independently reviewed by 2 reviewers. Disagreements on study inclusion or exclusion were resolved by consensus. Next, full-text articles of potentially eligible studies were retrieved for further assessment. Studies were included in the meta-analysis if they provided both sensitivity and specificity data of serum GP73 and AFP used for diagnosing HCC based on histopathological confirmation. Only English-language full-text articles were reviewed and included in the final analysis.

### Data extraction and quality assessment

Two reviewers independently extracted data such as first author name, year of acceptance for publication, country of study, number of patients with HCC, number of controls (healthy subjects or patients with cirrhosis, hepatitis, and other benign liver diseases), test methodology, cut-off value, and raw data for analyzing sensitivity and specificity (number of true-positive, false-positive, false-negative, and true-negative results) from the included studies. The quality of the included studies was assessed using the tool Quality Assessment of studies of Diagnostic Accuracy included in Systematic reviews (QUADAS; Cochrane Collaboration). Fourteen items in the QUADAS checklist were scored “yes”, “no”, or “unclear” [17].

### Data analysis

A heterogeneity test and a random-effect model were used in case of heterogeneity between the included studies, whereas a fixed-effect model was used in the absence of any heterogeneity. In this study, the following analyses were performed: Spearman correlation coefficient, threshold effect, and diagnostic odds ratio (DOR; used to eliminate possible threshold effect); the overall sensitivity, specificity, positive likelihood ratio (PLR), and negative likelihood ratio (NLR); summary receiver operating characteristic (SROC) curve analysis; area under the curve (AUC) analysis; meta-regression analysis; and publication bias analysis. Publication bias was determined using Stata (version 12.0), whereas all other analyses were performed using Meta-Disc (version 1.4).

## Results

### Study selection and analysis of study quality

**Fig 1:**
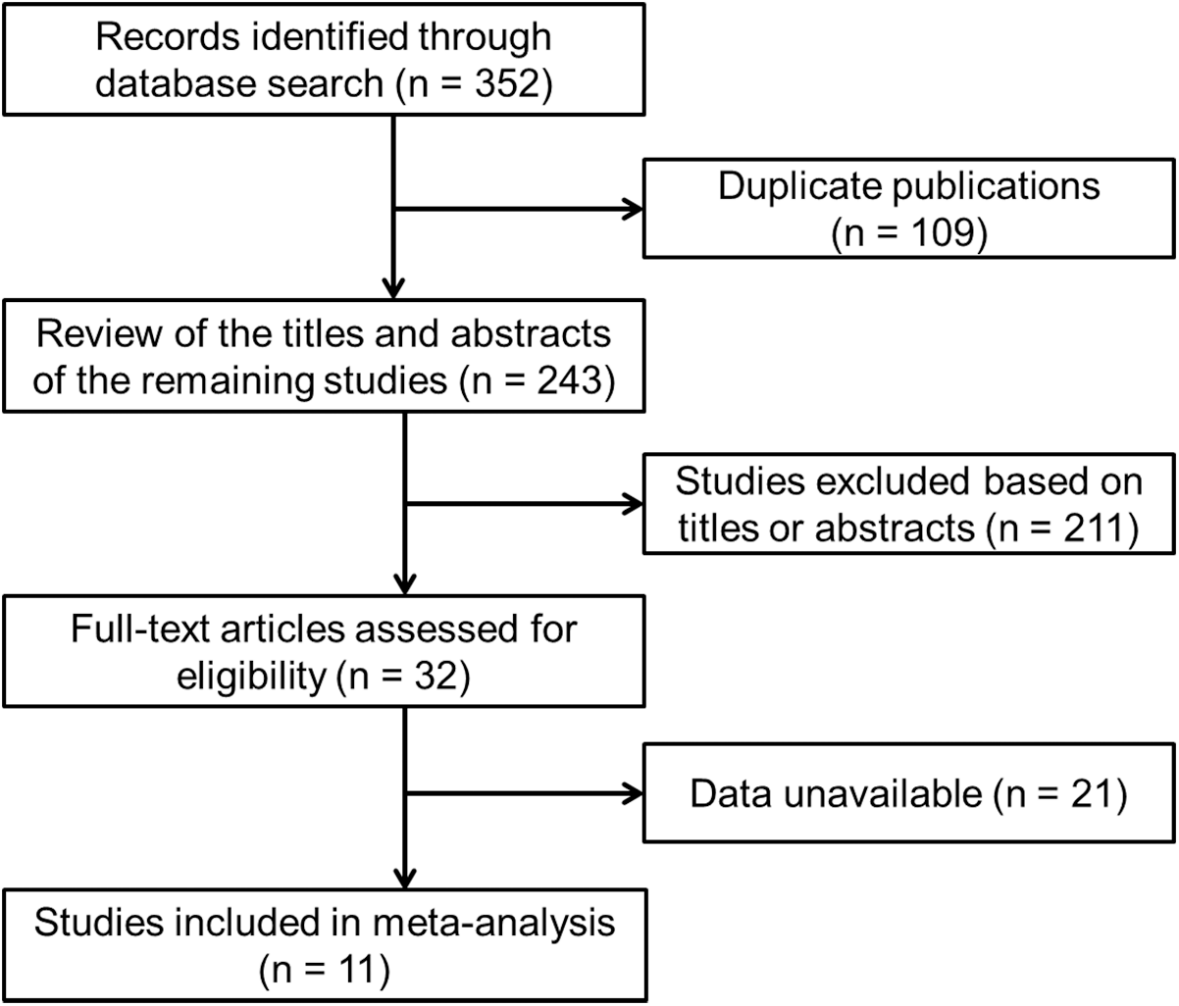
Flowchart of the study selection strategy.

The initial search identified 352 relevant studies, of which 109 were duplicates. After reviewing the titles and abstracts of the remaining 243 studies, 211 studies were excluded from the meta-analysis. The remaining 32 studies were considered eligible, and their full-text articles were reviewed. Of these, 21 studies were excluded because they were not diagnostic studies or they did not report sufficient data to construct a 2 × 2 table. Finally, 11 studies were included in the meta-analysis. The process of study selection is summarized in Fig 1. The 11 studies included 1764 patients with HCC and 4659 controls. All the patients underwent a single test for determining serum levels of GP73 and AFP [9,18–27]. Four of the 11 studies included 1025 patients with HCC and 3813 controls who also underwent a test to determine the serum levels of GP73 + AFP [9,19,20,24]. Characteristics of the included studies are listed in Table 1. The results of quality assessment of the 11 studies by using QUADAS are shown in Table 2. Summary scores were not calculated because their interpretation could be challenging and potentially misleading [28]. The details of the 14 items in QUADAS (Table 2) are the following:

1. The spectrum of patients in all the selected studies was representative of the patients who received the test in regular clinical practice. 2. All 11 studies clearly defined selection and exclusion criteria. 3. All studies used appropriate reference standards to accurately classify the target condition. 4. In 4 of the included studies, blood samples were collected before intervention, but this was unclear in the case of the others studies. 5. In 8 studies, patients were tested and their disease status was confirmed bases on the aforementioned reference standard, but this is unclear for the other studies. 6. In 2 studies, patients received the same reference standard regardless of the index test result. 7. In all 11 studies, the reference standard used was independent of the index test. 8. In 7 studies, the execution of the index test was described in sufficient detail to permit replication of the test. 9. In 7 studies, the execution of the reference standard was described in sufficient detail to permit its replication. 10. The index test results in none of the studies were interpreted without knowledge of the results of the reference standard. 11. In all studies, the reference standard results were interpreted without knowledge of the results of the index test. 12. In 10 studies, the same clinical data were available when test results were interpreted as would be available when the test is used in practice. 13. In 10 studies, uninterpretable or intermediate test results were reported. 14. In 10 studies, withdrawals from the study were explained.

**Table 1:** Main characteristics of the included studies.

First author,	Country	Patients with	GP73						AFP						GP73 + AFP			
year (ref.)		HCC/Controls	TP	FP	FN	TN	Cut-off	Method	TP	FP	FN	TN	Cut-off	Method	TP	FP	FN	TN
**Jia, 2014 [18]**	China	102/179	79	20	23	159	150 ng/mL	ELISA	53	39	49	140	400 ng/mL	Immunoassay	-	-	-	-
**Wang, 2013 [19]**	China	84/173	62	32	22	141	8.5 RU	Immunoblotting	66	26	22	147	NK	Immunoassay	67	26	17	147
**Hou, 2013 [20]**	China	79/105	58	21	21	84	78.1 ng/L	Immunoblotting	44	14	35	91	47.8 ng/mL	Immunoassay	70	32	9	73
**Shi, 2011 [9]**	China	73/107	50	7	23	100	100 μg/L	ELISA	21	1	52	106	400 μg/L	ELISA	54	8	19	99
**Morota, 2011 [21]**	Japan	70/159	62	61	8	98	94.7 μg/L	ELISA	44	13	26	146	15.3 μg/L	ELISA	-	-	-	-
**Tian, 2010 [22]**	China	153/95	115	46	38	49	113.89 μg/L	ELISA	145	50	8	45	13.6 μg/L	ELISA	-	-	-	-
**Ozkan, 2010 [23]**	Turkey	75/55	62	50	13	5	0.078 mg/mL	ELISA	51	3	24	52	13 ng/mL	ELISA	-	-	-	-
**Mao, 2010 [24]**	China/USA	789/3428	589	89	200	3339	8.5 RU	Immunoblotting	459	504	330	2924	35 ng/mL	Immunoassay	704	507	85	2921
**Hu, 2009 [25]**	China	31/93	24	15	7	78	7.4 RU	Western blotting	15	3	16	90	36 μg/L	ELISA	-	-	-	-
**Wang, 2009 [26]**	USA	164/113	156	64	8	49	NK	Immunoblotting	156	79	8	34	NK	ELISA	-	-	-	-
**Marrero, 2005 [27]**	USA	144/152	99	21	45	131	10 RU	Immunoblotting	43	6	101	146	99 ng/mL	ELISA	-	-	-	-

TP: true positive; FP: false positive; FN: false negative; TN: true negative; -: no data for this category; RU: relative unit; NK: not known; ref.: reference number; HCC: hepatocellular carcinoma.

**Table 2:** Quality assessment of the 11 studies by using QUADAS.

Item	1	2	3	4	5	6	7	8	9	10	11
**Representative patient spectrum?**	Y	Y	Y	Y	Y	Y	Y	Y	Y	Y	Y
**Selection criteria?**	Y	Y	Y	Y	Y	Y	Y	Y	Y	Y	Y
**Acceptable reference standard?**	Y	Y	Y	Y	Y	Y	Y	Y	Y	Y	Y
**Acceptable delay between tests?**	UC	UC	UC	UC	Y	UC	UC	Y	UC	Y	Y
**Partial verification avoided?**	UC	Y	Y	UC	UC	Y	Y	Y	Y	Y	Y
**Differential verification avoided?**	Y	UC	Y	UC	UC	N	N	N	N	N	N
**Incorporation avoided?**	Y	Y	Y	Y	Y	Y	Y	Y	Y	Y	Y
**Index test execution?**	Y	Y	Y	Y	UC	UC	Y	N	N	Y	Y
**Reference standard execution?**	Y	Y	N	N	N	Y	Y	Y	N	Y	Y
**Reference standard results blinded?**	N	N	N	N	N	N	N	N	N	N	N
**Index test results blinded?**	Y	Y	Y	Y	Y	Y	Y	Y	Y	Y	Y
**Relevant clinical information?**	Y	Y	Y	Y	UC	Y	Y	Y	Y	Y	Y
**Uninterpretable results reported?**	Y	Y	Y	UC	Y	Y	Y	Y	Y	Y	Y
**Withdrawals explained?**	Y	Y	Y	N	Y	Y	Y	Y	Y	Y	Y

Y: yes; N: no; UC: unclear; QUADAS: Quality Assessment of studies of Diagnostic Accuracy included in Systematic reviews.

### Summary diagnostic accuracy of GP73, AFP, and GP73 + AFP for diagnosing HCC

**Fig 2:**
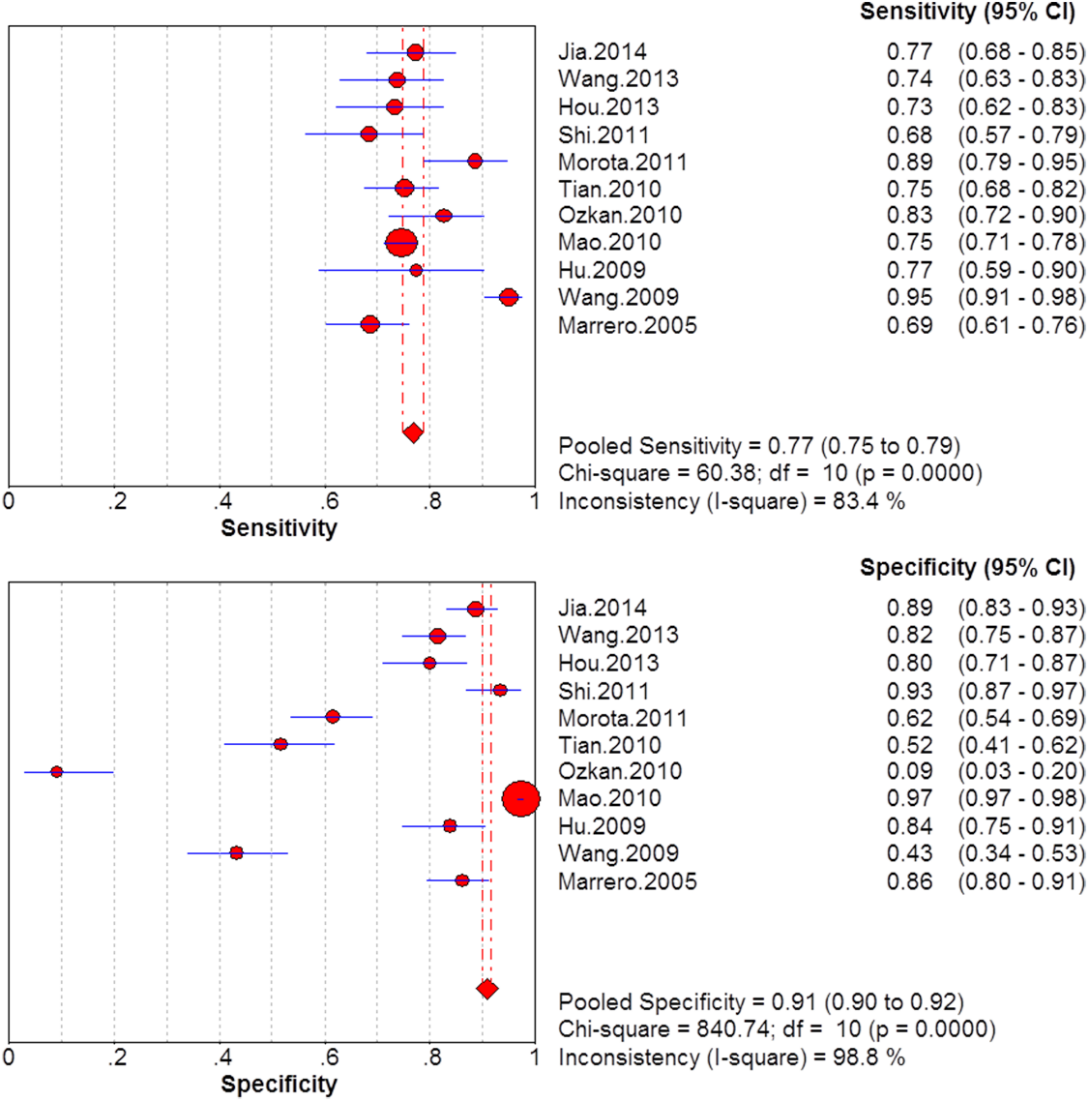
Forest plots of the sensitivity and specificity of GP73 for diagnosing HCC.

**Fig 3:**
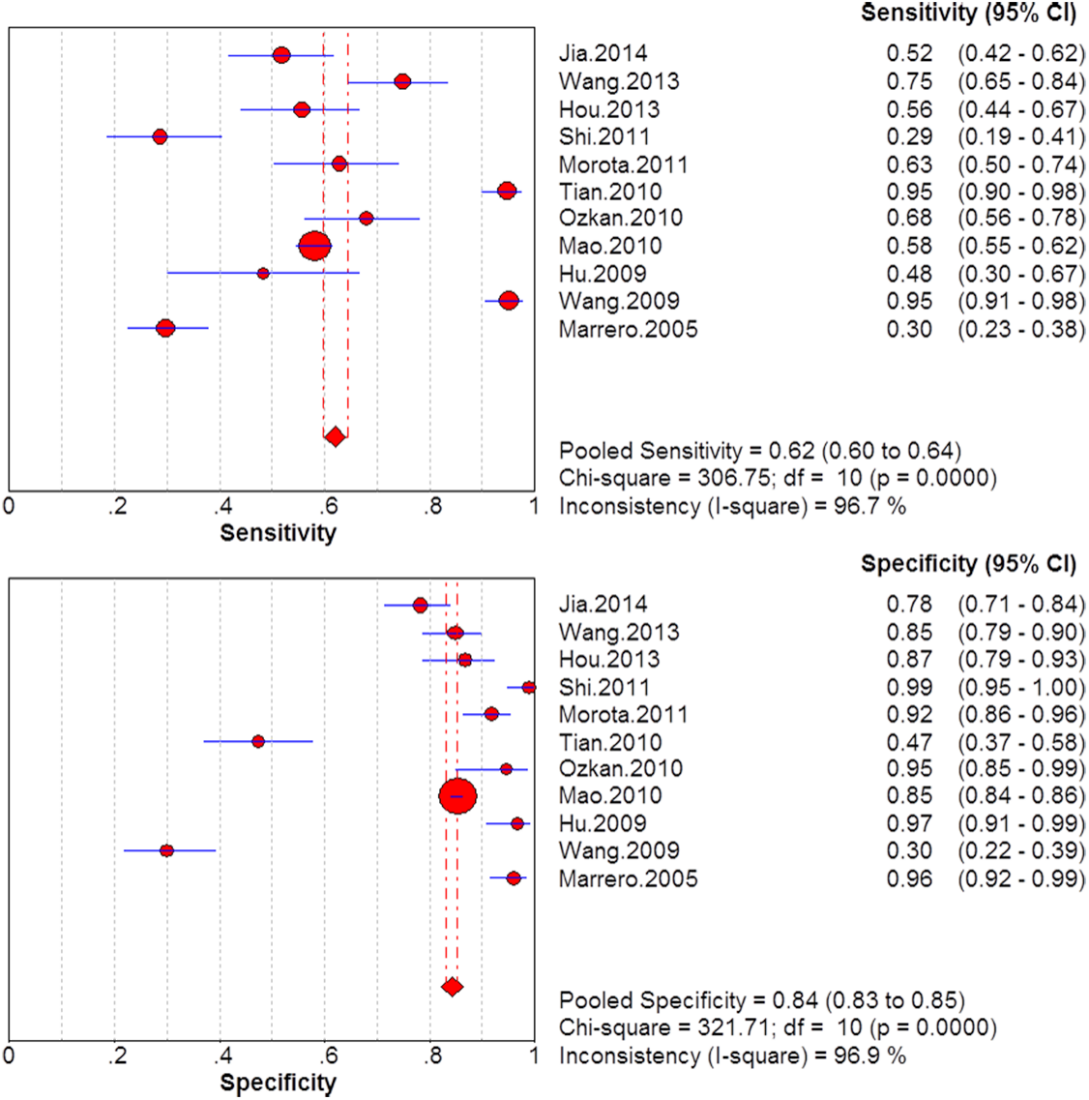
Forest plots of the sensitivity and specificity of AFP for diagnosing HCC.

**Fig 4:**
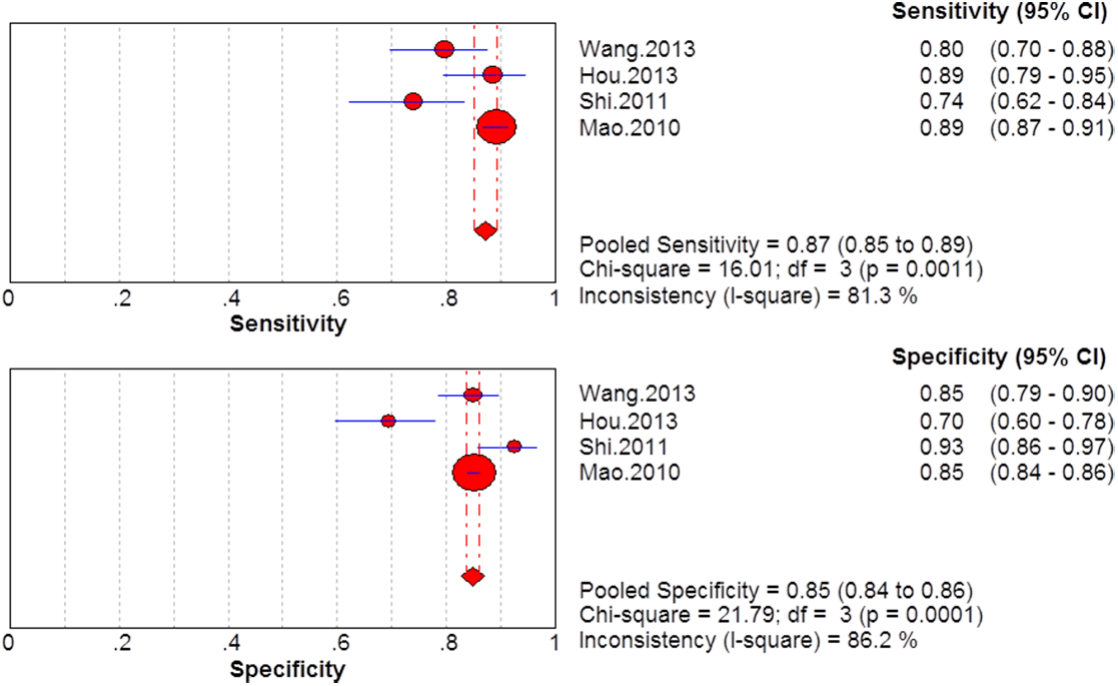
Forest plots of the sensitivity and specificity of GP73 + AFP for diagnosing HCC.

The sensitivity and specificity of GP73, AFP, and GP73 + AFP for diagnosing HCC are presented using forest plots in Figs 2–4, and the results show significant heterogeneity (sensitivity: I^2^ = 83.4%, 96.7%, and 81.3% for GP73, AFP and GP73 + AFP, respectively; specificity: I^2^ = 98.8%, 96.9%, and 86.2% for GP73, AFP, and GP73 + AFP, respectively). Therefore, the random-effects model was used for analysis. Pooled sensitivity and specificity were, respectively, 0.77 (95% CI: 0.75–0.79) and 0.91 (95% CI: 0.90–0.92) for GP73; 0.62 (95% CI: 0.60–0.64) and 0.84 (95% CI: 0.83–0.85) for AFP; and 0.87 (95% CI: 0.85–0.89) and 0.85 (95% CI: 0.84–0.86) for GP73 + AFP.

**Fig 5:**
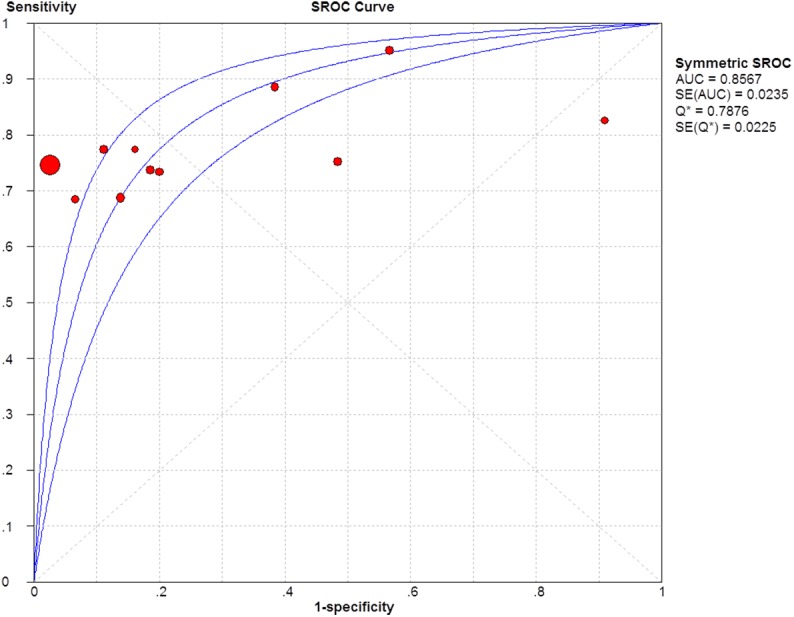
SROC curve of GP73 for diagnosing HCC.

**Fig 6:**
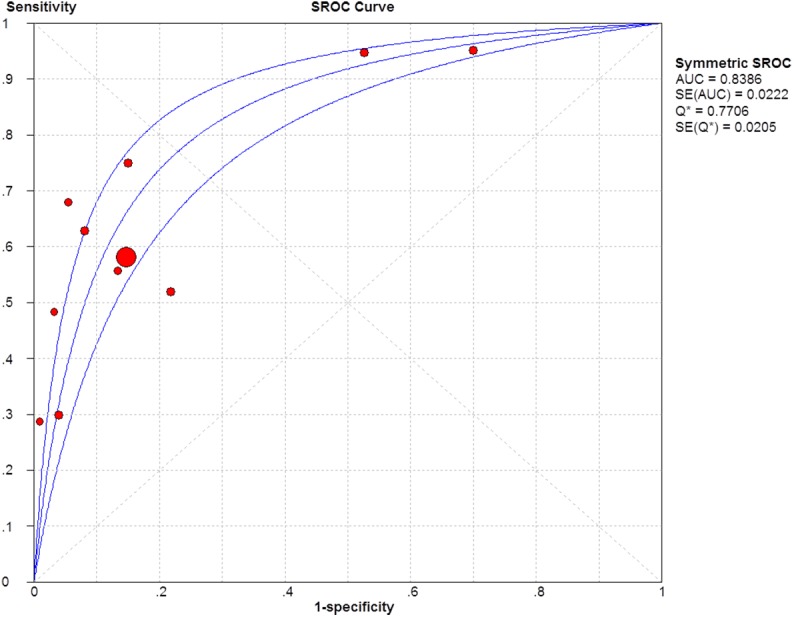
SROC curve of AFP for diagnosing HCC.

**Fig 7:**
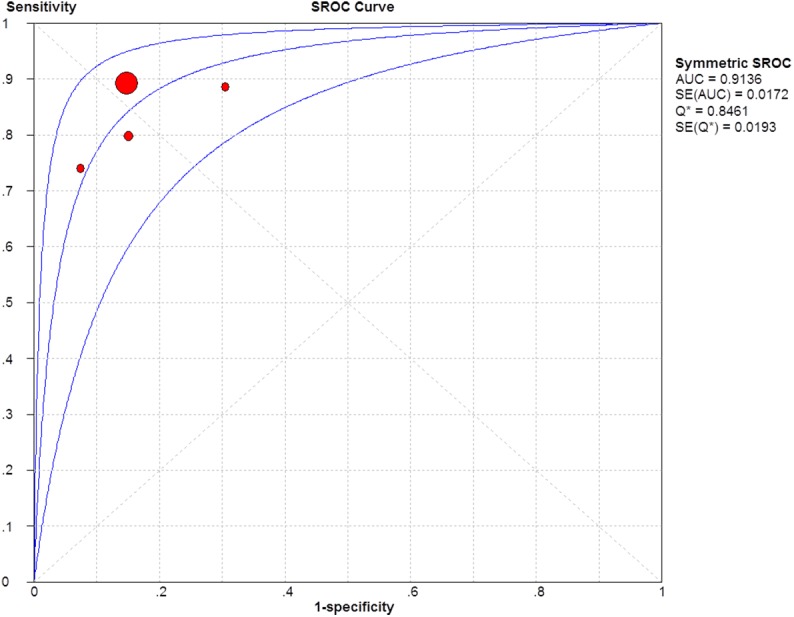
SROC curve of GP73 + AFP for diagnosing HCC.

The sensitivity and specificity of the studies were plotted using SROC curves (Figs 5–7). The AUC value was 0.86 for GP73, 0.84 for AFP, 0.91 for GP73 + AFP, which suggests that these biomarkers showed moderate accuracy in HCC diagnoses. The pooled PLR was 3.95 (95% CI: 1.69–9.26) for GP73, 4.48 (95% CI: 2.84–7.07) for AFP, and 5.22 (95% CI: 3.44–7.92) for GP73 + AFP, whereas the pooled NLR was 0.31 (95% CI: 0.25–0.39) for GP73, 0.44 (95% CI: 0.35–0.55) for AFP, and 0.19 (95% CI: 0.12–0.30) for GP73 + AFP.

The Spearman correlation coefficient for AFP was 0.773 (P = 0.005), which indicated a threshold effect. The threshold effect was considered as one of the reasons for heterogeneity, but other reasons for heterogeneity could not be determined. The Spearman correlation coefficients for GP73 and GP73 + AFP were 0.6 (P = 0.051) and 0.4 (P = 0.6), respectively, which indicated a contrasting result. Obtaining the DOR is a primary outcome method used for eliminating a possible threshold effect and for moderately differentiating between patients with and without cancer [29]. In this study, the DOR was highest for GP73 + AFP and lowest for AFP alone. These results are listed in Table 3.

**Table 3:** Summary of the diagnostic accuracy of GP73, AFP, and GP73 + AFP.

Marker	Pooled sensitivity	Pooled specificity	PLR	NLR	DOR	AUC
**GP73**	0.77 (0.75–0.79)	0.91 (0.90–0.92)	3.95 (1.69–9.26)	0.31 (0.25–0.39)	12.49 (4.91–31.79)	0.86
**AFP**	0.62 (0.60–0.64)	0.84 (0.83–0.85)	4.48 (2.84–7.07)	0.44 (0.35–0.55)	11.61 (8.02–16.81)	0.84
**GP73 + AFP**	0.87 (0.85–0.89)	0.85 (0.84–0.86)	5.22 (3.44–7.92)	0.19 (0.12–0.30)	30.63 (18.10–51.84)	0.91

95% confidence intervals are shown in brackets.

### Meta-regression analysis for heterogeneity and publication bias

**Fig 8:**
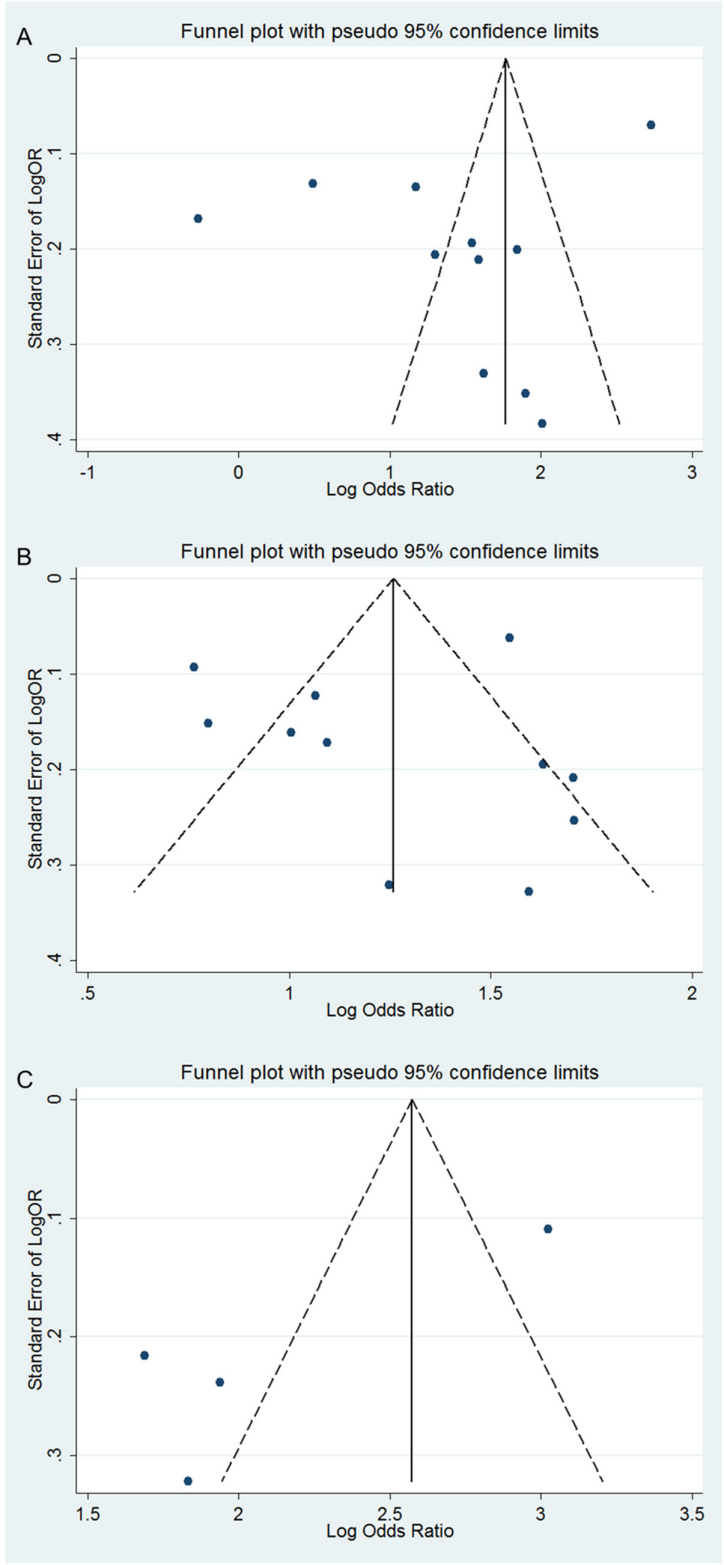
Deeks’ funnel plots of GP73, AFP, and GP73 + AFP for the studies included in the meta-analysis. A, GP73; B, AFP; and C, GP73 + AFP.

Significant heterogeneity was observed among the studies included in this meta-analysis. Because of the unsatisfactory quality of the test methodologies used in the included studies, the small number of studies, and incomplete data, only 3 covariates (year of acceptance for publication, country of study, and assay methodology) were included in meta-regression analysis to assess their impact on sensitivity and specificity. DOR is a commonly used accuracy measure because it measures a diagnostic performance that includes both sensitivity and specificity or both PLR and NLR. As a global measure of diagnostic accuracy, DOR is suitable for comparing the summary diagnostic accuracy of distinct tests [30]. We observed that the 3 covariates of GP73 and AFP did not exert a statistically significant effect on DOR (Table 4). The number of studies that provided the sensitivity and specificity data of GP73 + AFP for diagnosing HCC was too small to allow a meta-regression analysis for heterogeneity. However, for publication bias analysis, funnel plots were obtained using the metafunnel command of Stata version 12.0 (Fig 8).

**Table 4:** Meta-regression analysis of the effects of GP73 and AFP on diagnostic accuracy.

Variable	GP73					AFP				
	Coeff.	Std. Err.	P value	RDOR	95% CI	Coeff.	Std. Err.	P value	RDOR	95% CI
**Year**	0.243	0.1493	0.1549	1.27	0.88–1.84	-0.021	0.1949	0.9169	0.98	0.61–1.58
**Country**	0.430	0.2198	0.0983	1.54	0.90–2.63	-0.146	0.2206	0.5332	0.86	0.50–1.48
**Method**	0.329	0.4321	0.4756	1.39	0.48–4.00	0.847	0.6313	0.2280	2.33	0.50–10.94

RDOR: ratio of diagnostic odds ratio.

## Discussion

HCC is a major public health concern worldwide. Early detection of HCC is critical for accurate treatment and for improving the health and survival of patients. In this meta-analysis involving 11 studies, we evaluated the accuracy of serum GP73, AFP, and GP73 + AFP for diagnosing HCC. However, because of several methodological limitations, the diagnostic accuracy values reported in the 11 studies showed significant heterogeneity. Whereas 4 of the 11 studies reported that GP73 was superior to AFP as a serum biomarker of HCC [18,24,25,27], the remaining 7 studies reported contrasting or ambiguous results [9,19–23,26]. Furthermore, although 4 of the 11 studies reported the accuracy of GP73 + AFP for diagnosing HCC, the conclusions of these studies were inconsistent [9,19,20,24]. Eight of the 11 studies included in our meta-analysis [9,21–27] were also included in a 2012 study by Zhou et al, who compared the accuracy of GP73 with that of AFP for diagnosing HCC [31]. However, our meta-analysis evaluated the accuracy of GP73 + AFP for diagnosing HCC. Moreover, we included 3 additional studies to increase the credibility of the results obtained for the diagnostic accuracy of GP73 + AFP. GP73, a potential serum biomarker of HCC, is a 73-kDa transmembrane glycoprotein containing 400 amino acids that is normally expressed in epithelial cells of various human tissues [11]. High levels of serum GP73 were first reported by Block et al in 2005 in patients with hepatitis B virus-associated HCC [10]. In the same year, Marrero et al [27] confirmed that levels of serum GP73 in patients with HCC were considerably higher than those in patients with cirrhosis, and additionally reported that the sensitivity of GP73 for the early diagnosis of HCC was superior to that of AFP. Moreover, the systematic review and meta-analysis of Witjes et al [32] further confirmed these results. Since these findings were published, increasing numbers of studies have been performed on GP73 as a biomarker of HCC.

Our meta-analysis showed that pooled sensitivity and specificity of GP73 were higher than those of AFP, and that these values for GP73 + AFP were higher than those for GP73 or AFP alone. The pooled PLRs of GP73, AFP, and GP73 + AFP were 3.95, 4.48, and 5.22, respectively, whereas the pooled NLRs were 0.31, 0.44 and 0.19, respectively. A high PLR value indicates a high chance of diagnosing HCC, and a low NLR value indicates a high ability of the diagnostic methods to exclude non-HCC diseases. Thus, for a diagnostic test, a high PLR value indicates superior performance, whereas a high NLR value indicates poor performance. In this study, the PLR and NLR values of GP73 were lower than those of AFP. By contrast, the PLR value of GP73 + AFP was higher than that of GP73 or AFP alone and its NLR value was lower than that of GP73 or AFP alone. These results indicated that the diagnostic accuracy of GP73 was comparable to that of AFP, but that the diagnostic accuracy of GP73 + AFP was superior to that of GP73 or AFP alone.

DOR converts the strengths of sensitivity and specificity into a single index that represents diagnostic accuracy. DOR is defined as the ratio of the odds of positive test results of participants with a disease to the odds of positive test results of participants without that disease [33]. DOR values range from 0 to infinity, with a higher value indicating higher accuracy. In this meta-analysis, the mean DOR values of GP73, AFP, and GP73 + AFP were 12.49, 11.61, and 30.63, respectively; this suggests that serum levels of GP73 + AFP were more helpful for early diagnosis of HCC than were the serum levels of GP73 or AFP alone, and that the accuracies of GP73 and AFP alone for diagnosing HCC did not differ markedly.

The SROC curve and AUC are important for assessing diagnostic data in meta-analyses. In SROC curve analysis, the emphasis is on a comprehensive evaluation of a diagnostic method and not on simply the method’s sensitivity or specificity [34–36]. The AUC is a useful and widely used index of the SROC curve in meta-analyses and it ranges from 1, which indicates a perfect test that correctly classifies all cases and non-cases, to 0, which indicates a test that does not perform an accurate diagnosis. The AUC also shows extremely steady performance in heterogeneity tests. In our meta-analysis, the AUC values of GP73, AFP, and GP73 + AFP were 0.86, 0.84, and 0.91, respectively, which indicates that serum levels of GP73 + AFP showed higher accuracy in HCC diagnosis than did the serum levels of GP73 or AFP alone. Moreover, these AUC values indicated that the diagnostic accuracy of GP73 was superior to that of AFP.

In a meta-analysis, one of the major goals is to analyze the reasons for heterogeneity rather than to compute a single summary measure. An I^2^ value of >50% indicates significant heterogeneity [37]. Here, I^2^ values of the sensitivity and specificity of GP73, AFP, and GP73 + AFP are presented using forest plots, and these reveal significant heterogeneity. A threshold effect was only one of the reasons for heterogeneity, and the meta-regression analysis for heterogeneity performed in this study showed no statistical difference. Therefore, our meta-analysis could not determine all the reasons responsible for the heterogeneity observed among the included studies. This might be because of inconsistencies in the assessment of study quality and the availability of limited data and incomplete information. Asymmetrical funnel plots indicated publication bias, which might exist because of diverse reasons such as poor methodological quality of small studies, inclusion of numerous studies without registration, true heterogeneity, artifactual results, and other causes. Therefore, several recent studies have not evaluated publication bias among studies that assessed the diagnostic accuracy of distinct markers [34,38,39]. In this study, we also repeated all of these pooled sensitivity and specificity calculations with each of the 11 studies removed individually, and found that this did not markedly affect the final conclusion. These findings clearly reflect the credibility and stability of the results of this meta-analysis.

In conclusion, the results of this meta-analysis showed that serum GP73 + AFP exhibited significantly higher diagnostic accuracy for HCC than did serum GP73 or AFP alone. Moreover, the accuracy of GP73 for diagnosing HCC was superior to that of AFP, which was similar to that observed in the Zhou et al study [31]. However, the number of studies that evaluated the serum levels of both GP73 and AFP was too small to allow meta-regression analysis for heterogeneity, and data on early detection of HCC were lacking; thus, further investigation must be conducted to assess the accuracy of serum GP73 + AFP for the early diagnosis of HCC. Moreover, additional biomarkers should be combined with GP73 and AFP for comprehensive diagnosis of HCC.

## Supporting Information

S1 File Original data of this meta-analysis. (XLSX)

S2 File Original output of Meta-disc and Stata software. (DOCX)

## References

[1] Siegel R, Naishadham D, Jemal A. Cancer statistics, 2012. CA Cancer J Clin. 2012;62: 10–29. 10.3322/caac.20138 22237781

[2] Thun MJ, DeLancey JO, Center MM, Jemal A, Ward EM. The global burden of cancer: priorities for prevention. Carcinogenesis. 2010;31: 100–110. 10.1093/carcin/bgp263 19934210 PMC2802672

[3] Njei B, Rotman Y, Ditah I, Lim JK. Emerging trends in hepatocellular carcinoma incidence and mortality. Hepatology. 2015;61: 191–199. 10.1002/hep.27388 25142309 PMC4823645

[4] Zhang ZM, Zhang YM, Yao F, Yi P, Huang S, Liu JY, et al Analysis on postoperative efficacy of radical hepatectomy for patients with non-HBV/HCV hepatocellular carcinoma. Asian Pac J Cancer Prev. 2015;16: 3479–3483. 25921165 10.7314/apjcp.2015.16.8.3479

[5] Lu FM, Zhuang H. Management of hepatitis B in China. Chin Med J. 2009;122: 3–4. 19187608

[6] Ertle JM, Heider D, Wichert M, Keller B, Kueper R, Hilgard P, et al A combination of α-fetoprotein and des-γ-carboxy prothrombin is superior in detection of hepatocellular carcinoma. Digestion. 2013;87: 121–131. 10.1159/000346080 23406785

[7] Yamamoto K, Imamura H, Matsuyama Y, Hasegawa K, Beck Y, Sugawara Y, et al Significance of alpha-fetoprotein and des-gamma-carboxy prothrombin in patients with hepatocellular carcinoma undergoing hepatectomy. Ann Surg Oncol. 2009;16: 2795–2804. 10.1245/s10434-009-0618-y 19669841

[8] Morimoto M, Numata K, Nozaki A, Kondo M, Moriya S, Taguri M, et al Novel lens culinaris agglutinin-reactive fraction of α-fetoprotein: a biomarker of hepatocellular carcinoma recurrence in patients with low α-fetoprotein concentrations. Int J Clin Oncol. 2012;17: 373–379. 10.1007/s10147-011-0306-3 21847534

[9] Shi Y, Chen J, Li L, Sun Z, Zen L, Xu S, et al A study of diagnostic value of Golgi protein GP73 and its genetic assay in primary hepatic carcinoma. Technol Cancer Res Treat. 2011;10: 287–294. 21517136 10.7785/tcrt.2012.500205

[10] Block TM, Comunale MA, Lowman M, Steel LF, Romano PR, Fimmel C, et al Use of targeted glycoproteins to identify serum glycoproteins that correlated with liver cancer in woodchucks and humans. Proc Natl Acad Sci USA. 2005;102: 779–784. 15642945 10.1073/pnas.0408928102 PMC545516

[11] Kladney RD, Bulla GA, Guo L, Mason AL, Tollefson AE, Simon DJ, et al GP73, a novel Golgi-localized protein upregulated by viral infection. Gene. 2000;249: 53–65. 10831838 10.1016/S0378-1119(00)00136-0 PMC7127640

[12] Kladney RD, Cui X, Bulla GA, Brunt EM, Fimmel CJ. Expression of GP73, a resident Golgi membrane protein, in viral and nonviral liver disease. Hepatology. 2002;35: 1431–1440. 12029628 10.1053/jhep.2002.32525

[13] Schwegler EE, Cazares L, Steel LF, Adam BL, Johnson DA, Semmes OJ, et al SELDI‑TOF MS profiling of serum for detection of the progression of chronic hepatitis C to hepatocellular carcinoma. Hepatology. 2005;41: 634–642. 15726646 10.1002/hep.20577

[14] Iftikhar R, Kladney RD, Havlioglu N, Schmitt-Gräff A, Gusmirovic I, Solomon H, et al Disease- and cell-specific expression of GP73 in human liver disease. Am J Gastroenterol. 2004;99: 1087–1095. 15180730 10.1111/j.1572-0241.2004.30572.x

[15] Maitra A, Thuluvgth PJ. GP73 and liver disease: a (Golgi) complex enigma. Am J Gastroenterol. 2004;99: 1096–1098. 15180731 10.1111/j.1572-0241.2004.40410.x

[16] Ma C, Zhang Q, Qu J, Zhao X, Li X, Liu Y, et al A precise approach in large scale core-fucosylated glycoprotein identification with low- and high-normalized collision energy. J Proteomics. 2015;114: 61–70. 10.1016/j.jprot.2014.09.001 25220145

[17] Whiting P, Rutjes AW, Reitsma JB, Bossuyt PM, Kleijnen J. The development of QUADAS: a tool for the quality assessment of studies of diagnostic accuracy included in systematic reviews. BMC Med Res Methodol. 2003;10: 3–25. 10.1186/1471-2288-3-25 PMC305345 14606960

[18] Jia Z, Wang L, Liu C, Yu Z, Chai L, Zhao M. Evaluation of α-fetoprotein-L3 and Golgi protein 73 detection in diagnosis of hepatocellular carcinoma. Contemp Oncol (Pozn). 2014;18: 192–196. 25520580 10.5114/wo.2014.43157 PMC4268990

[19] Wang Y, Yang H, Xu H, Lu X, Sang X, Zhong S, et al Golgi protein 73, not glypican–3, may be a tumor marker complementary to α-fetoprotein for hepatocellular carcinoma diagnosis. J Gastroenterol Hepatol. 2014;29: 597–602. 10.1111/jgh.12461 24236824

[20] Hou SC, Xiao MB, Ni RZ, Ni WK, Jiang F, Li XY, et al Serum GP73 is complementary to AFP and GGT-II for the diagnosis of hepatocellular carcinoma. Oncol Lett. 2013;6: 1152–1158. 24137480 10.3892/ol.2013.1522 PMC3796428

[21] Morota K, Nakagawa M, Sekiya R, Hemken PM, Sokoll LJ, Elliott D, et al A comparative evaluation of Golgi protein–73, fucosylated hemopexin, α-fetoprotein, and PIVKA-II in the serum of patients with chronic hepatitis, cirrhosis, and hepatocellular carcinoma. Clin Chem Lab Med. 2011;49: 711–718. 10.1515/CCLM.2011.097 21231906

[22] Tian L, Wang Y, Xu D, Gui J, Jia X, Tong H, et al Serological AFP/Golgi protein 73 could be a new diagnostic parameter of hepatic diseases. Int J Cancer. 2011;129: 1923–1931. 10.1002/ijc.25838 21140449

[23] Ozkan H, Erdal H, Tutkak H, Karaeren Z, Yakut M, Yüksel O, et al Diagnostic and prognostic validity of Golgi protein 73 in hepatocellular carcinoma. Digestion. 2011;83: 83–88. 10.1159/000320379 21042019

[24] Mao Y, Yang H, Xu H, Lu X, Sang X, Du S, et al Golgi protein 73 (GOLPH2) is a valuable serum marker for hepatocellular carcinoma. Gut. 2010;59: 1687–1693. 10.1136/gut.2010.214916 20876776

[25] Hu JS, Wu DW, Liang S, Miao XY. GP73, a resident Golgi glycoprotein, is sensibility and specificity for hepatocellular carcinoma of diagnosis in a hepatitis B-endemic Asian population. Med Oncol. 2010;27: 339–345. 10.1007/s12032-009-9215-y 19399652

[26] Wang M, Long RE, Comunale MA, Junaidi O, Marrero J, Di Bisceglie AM, et al Novel fucosylated biomarkers for the early detection of hepatocellular carcinoma. Cancer Epidemiol Biomarkers Prev. 2009;18: 1914–1921. 10.1158/1055-9965.EPI-08-0980 19454616 PMC4413450

[27] Marrero JA, Romano PR, Nikolaeva O, Steel L, Mehta A, Fimmel CJ, et al GP73, a resident Golgi glycoprotein, is a novel serum marker for hepatocellular carcinoma. J Hepatol. 2005;43: 1007–1012. 16137783 10.1016/j.jhep.2005.05.028

[28] Whiting P, Harbord R, Kleijnen J. No role for quality scores in systematic reviews of diagnostic accuracy studies. BMC Med Res Methodol. 2005;5: 19 15918898 10.1186/1471-2288-5-19 PMC1184082

[29] Walter SD. Properties of the summary receiver operating characteristic (SROC) curve for diagnostic test data. Stat Med. 2002;21: 1237–1256. 12111876 10.1002/sim.1099

[30] Lijmer JG, Bossuyt PM, Heisterkamp SH. Exploring sources of heterogeneity in systematic reviews of diagnostic tests. Stat Med. 2002;21: 1525–1537. 12111918 10.1002/sim.1185

[31] Zhou Y, Yin X, Ying J, Zhang B. Golgi protein 73 versus alpha-fetoprotein as a biomarker for hepatocellular carcinoma: a diagnostic meta-analysis. BMC Cancer. 2012;16: 12–17. 10.1186/1471-2407-12-17 PMC3292967 22244200

[32] Witjes CD, van Aalten SM, Steyerberg EW, Borsboom GJ, de Man RA, Verhoef C, et al Recently introduced biomarkers for screening of hepatocellular carcinoma: a systematic review and meta-analysis. Hepatol Int. 2013;7: 59–64. 10.1007/s12072-012-9374-3 23519638 PMC3601272

[33] Duval S, Tweedie R. Trim and fill: a simple funnel-plot based method of testing and adjusting for publication bias in meta-analysis. Biometrics. 2000;56: 455–463. 10877304 10.1111/j.0006-341x.2000.00455.x

[34] Yang J, Li J, Dai W, Wang F, Shen M, Chen K, et al Golgi protein 73 as a biomarker for hepatocellular carcinoma: a diagnostic meta-analysis. Exp Ther Med. 2015;9: 1413–1420. 25780444 10.3892/etm.2015.2231 PMC4353736

[35] Li YW, Chen ZG, Wang JC, Zhang ZM. Superparamagnetic iron oxide-enhanced magnetic resonance imaging for focal hepatic lesions: systematic review and meta-analysis. World J Gastroenterol. 2015;21: 4334–4344. 10.3748/wjg.v21.i14.4334 25892885 PMC4394096

[36] Jia X, Liu J, Gao Y, Huang Y, Du Z. Diagnosis accuracy of serum glypican–3 in patients with hepatocellular carcinoma: a systematic review with meta-analysis. Arch Med Res. 2014;45: 580–588. 10.1016/j.arcmed.2014.11.002 25446613

[37] Higgins JP, Thompson SG. Quantifying heterogeneity in a meta-analysis. Stat Med. 2002;21: 1539–1558. 12111919 10.1002/sim.1186

[38] Deeks JJ, Macaskill P, Irwig L. The performance of tests of publication bias and other sample size effects in systematic reviews of diagnostic test accuracy was assessed. J Clin Epidemiol. 2005;58: 882–893. 16085191 10.1016/j.jclinepi.2005.01.016

[39] Leeflang MM, Deeks JJ, Gatsonis C, Bossuyt PM. Cochrane diagnostic test accuracy working group: systematic reviews of diagnostic test accuracy. Ann Intern Med. 2008;149: 889–897. 19075208 10.7326/0003-4819-149-12-200812160-00008 PMC2956514

