# Supplementary material for: Diagnostic Value of the Combination of Golgi Protein 73 and Alpha-Fetoprotein in Hepatocellular Carcinoma: A Meta-Analysis
Source: PLoS One. 2015 Oct 6;10(10):e0140067. doi: 10.1371/journal.pone.0140067 (PMC4595485; doi:10.1371/journal.pone.0140067)
Supplement: S2 File — (DOCX) [file pone.0140067.s002.docx]

**Metadisc Software**

**GP73**

**Analysis of Diagnostic Threshold**

--------------------------------------------------------------------------------

Spearman correlation coefficient: 0.600 p-value= 0.051

(Logit(TPR) vs Logit(FPR)

--------------------------------------------------------------------------------

Moses' model (D = a + bS)

Weighted regression (Inverse Variance)

Var Coeff. Std. Error T p-value

--------------------------------------------------------------------------------

a 2.621 0.269 9.730 0.0000

b( 1) -0.520 0.142 3.653 0.0053

--------------------------------------------------------------------------------

Tau-squared estimate = 0.6545 (Convergence is achieved after 5 iterations)

Restricted Maximum Likelihood estimation (REML)

No. studies = 11

Filter OFF

Add 1/2 to all cells of the studies with zero

-----------------------------------------------------------------------------------------

**Meta-Regression(Inverse Variance weights)**

Var Coeff. Std. Err. p - value RDOR [95%CI]

-----------------------------------------------------------------------------------------

Cte. -1.448 2.1777 0.5308 ---- ----

S -0.526 0.1369 0.0085 ---- ----

year 0.243 0.1493 0.1549 1.27 (0.88;1.84)

country 0.430 0.2198 0.0983 1.54 (0.90;2.63)

method 0.329 0.4321 0.4756 1.39 (0.48;4.00)

-----------------------------------------------------------------------------------------

Tau-squared estimate = 0.5095 (Convergence is achieved after 9 iterations)

Restricted Maximum Likelihood estimation (REML)

No. studies = 11

Filter OFF

Add 1/2 to all cells of the studies with zero

**AFP**

**Analysis of Diagnostic Threshold**

--------------------------------------------------------------------------------

Spearman correlation coefficient: 0.773 p-value= 0.005

(Logit(TPR) vs Logit(FPR)

--------------------------------------------------------------------------------

Moses' model (D = a + bS)

Weighted regression (Inverse Variance)

Var Coeff. Std. Error T p-value

--------------------------------------------------------------------------------

a 2.423 0.232 10.436 0.0000

b( 1) -0.061 0.089 0.679 0.5144

--------------------------------------------------------------------------------

Tau-squared estimate = 0.3159 (Convergence is achieved after 6 iterations)

Restricted Maximum Likelihood estimation (REML)

No. studies = 11

Filter OFF

Add 1/2 to all cells of the studies with zero

-----------------------------------------------------------------------------------------

**Meta-Regression(Inverse Variance weights)**

Var Coeff. Std. Err. p - value RDOR [95%CI]

-----------------------------------------------------------------------------------------

Cte. 1.693 3.3480 0.6311 ---- ----

S -0.064 0.0928 0.5140 ---- ----

year -0.021 0.1949 0.9169 0.98 (0.61;1.58)

country -0.146 0.2206 0.5332 0.86 (0.50;1.48)

method 0.847 0.6313 0.2280 2.33 (0.50;10.94)

-----------------------------------------------------------------------------------------

Tau-squared estimate = 0.2415 (Convergence is achieved after 7 iterations)

Restricted Maximum Likelihood estimation (REML)

No. studies = 11

Filter OFF

Add 1/2 to all cells of the studies with zero

**GP73+AFP**

**Analysis of Diagnostic Threshold**

--------------------------------------------------------------------------------

Spearman correlation coefficient: 0.400 p-value= 0.600

(Logit(TPR) vs Logit(FPR)

--------------------------------------------------------------------------------

Moses' model (D = a + bS)

Weighted regression (Inverse Variance)

Var Coeff. Std. Error T p-value

--------------------------------------------------------------------------------

a 3.408 0.296 11.515 0.0075

b( 1) -0.121 0.326 0.371 0.7463

--------------------------------------------------------------------------------

Tau-squared estimate = 0.2388 (Convergence is achieved after 7 iterations)

Restricted Maximum Likelihood estimation (REML)

No. studies = 4

Filter OFF

Add 1/2 to all cells of the studies with zero

-----------------------------------------------------------------------------------------

**Meta-Regression(Inverse Variance weights)**

Var Coeff. Std. Err. p - value RDOR [95%CI]

-----------------------------------------------------------------------------------------

Cte. 6.759 1.0473 0.0979 ---- ----

S -0.046 0.2061 0.8599 ---- ----

year -0.288 0.0978 0.2083 0.75 (0.22;2.60)

-----------------------------------------------------------------------------------------

Tau-squared estimate = 0.0000 (Convergence is achieved after 1 iterations)

Restricted Maximum Likelihood estimation (REML)

No. studies = 4

Filter OFF

Add 1/2 to all cells of the studies with zero

-----------------------------------------------------------------------------------------

**Meta-Regression(Inverse Variance weights)**

Var Coeff. Std. Err. p - value RDOR [95%CI]

-----------------------------------------------------------------------------------------

Cte. 2.833 0.3163 0.0708 ---- ----

S -0.238 0.2238 0.4798 ---- ----

country 0.280 0.0950 0.2084 1.32 (0.40;4.42)

-----------------------------------------------------------------------------------------

Tau-squared estimate = 0.0000 (Convergence is achieved after 1 iterations)

Restricted Maximum Likelihood estimation (REML)

No. studies = 4

Filter OFF

Add 1/2 to all cells of the studies with zero

**STATA Software**

**GP73**

**
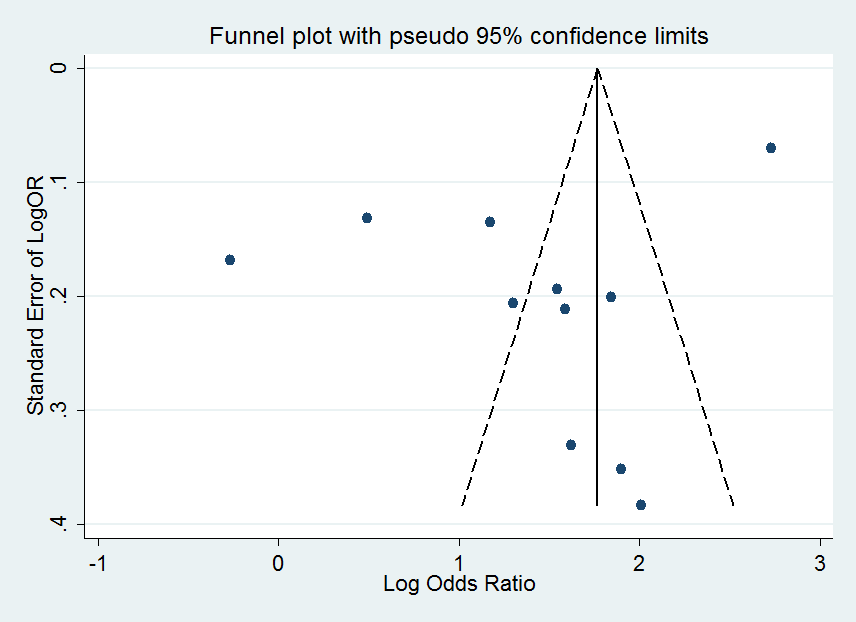
**

**AFP**

**
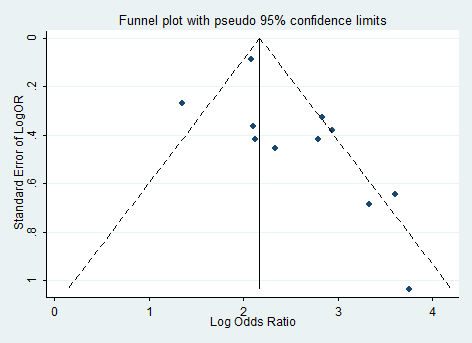
**

**GP73+AFP**

**
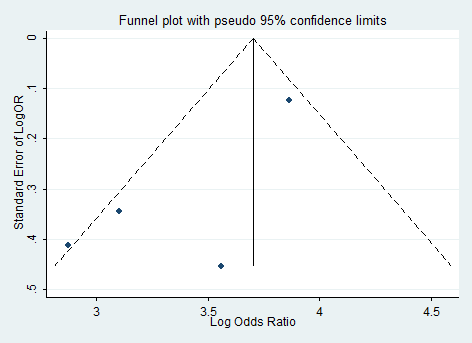
**
